# Supplementary material for: Neural network-based image reconstruction in swept-source optical coherence tomography using undersampled spectral data
Source: Light Sci Appl. 2021 Jul 29;10:155. doi: 10.1038/s41377-021-00594-7 (PMC8322159; doi:10.1038/s41377-021-00594-7)
Supplement: Supplementary file 1 — Supplementary Information [file 41377_2021_594_MOESM1_ESM.pdf]

Supplementary Information for

Neural network-based image reconstruction in swept-source optical coherence tomography using undersampled spectral data

Yijie Zhang[1,2,3†], Tairan Liu[1,2,3†], Manmohan Singh[4], Ege Çetintaş[1,2,3], Yilin Luo[1,2,3], Yair Rivenson[1,2,3], Kirill V. Larin[4,5], and Aydogan Ozcan[1,2,3,6,*]

[1]Electrical and Computer Engineering Department, University of California, Los Angeles, CA 90095, USA
[2]Department of Bioengineering, University of California, Los Angeles, CA 90095, USA
[3]California NanoSystems Institute, University of California, Los Angeles, CA 90095, USA
[4]Department of Biomedical Engineering, University of Houston, Houston, TX 77204, USA
[5]Department of Molecular Physiology and Biophysics, Baylor College of Medicine, University of Houston, Houston, TX 90095, USA
[6]Department of Surgery, David Geffen School of Medicine, University of California, Los Angeles, CA 90095, USA

*Correspondence: ozcan@ucla.edu
[†]Equally contributing authors

Supplementary Methods

DL-OCT on undersampled squeezed spectral data

All pre-processing methods for DL-OCT reported in the main text either interpolate or zero-pad the undersampled spectral data to its original size (before the undersampling). To further speed up the pre-processing step needed to feed image data to the reconstruction neural network, here we investigate the use of undersampled spectral data without any interpolation/padding steps.

After the $m\times$ spectral undersampling (e.g., $m = 2$ or 3), we squeeze the spectral data to $1/m$ of its original size without any interpolation/padding. Then, we apply a Fast Fourier transform (FFT) to this squeezed spectral data to obtain the undersampled OCT image. One example field-of-view resulting from this step is shown in Fig. S1(a-c). Next, we apply a combination of simple spatial transforms to convert the undersampled squeezed OCT images into a form that is equivalent to the undersampled OCT images processed with zero interpolation (shown in Fig. S1(d-e)). Using the undersampled squeezed A-line signal $g[n]$ of length N, the zero interpolated signal $g_0[n]$ with length mN can be represented as:

$$g_0[n] = \begin{cases} g[n/m] & \text{for } n \bmod m = 0 \\ 0 & \text{otherwise} \end{cases} \tag{S1}$$

The DFT of the $g_0[n]$ can be formulated as:

$$\begin{aligned} \mathrm{DFT}\left(g_0[n]\right)[k] &\equiv \sum_{n=0}^{mN-1} g_0[n] e^{-i2\pi\frac{n}{mN}k}, \text{for } k \in [0, mN-1] \\ &= \sum_{n=0}^{N-1} g[n] e^{-i2\pi\frac{n}{N}k}, \text{for } k \in [0, mN-1] \end{aligned} \tag{S2}$$

Specifically, for $k \in [pN, (p+1)N\text{-}1]$, where $0 \leqslant p \leqslant m\text{-}1$, we have:

$$\begin{aligned} \mathrm{DFT}\left(g_0[n]\right)[k] &= \sum_{n=0}^{N-1} g[n] e^{-i2\pi\frac{n}{N}k}, \text{for } k \in [pN, (p+1)N-1] \\ &= \sum_{n=0}^{N-1} g[n] e^{-i2\pi\frac{n}{N}(pN+k')}, \text{for } k' \in [0, N-1] \\ &= \sum_{n=0}^{N-1} g[n] e^{-i2\pi\frac{n}{N}k'}, \text{for } k' \in [0, N-1] \\ &\equiv \mathrm{DFT}\left(g[n]\right)[k], \text{for } k \in [0, N-1] \end{aligned} \tag{S3}$$

Equation S3 shows that the DFT of $m\times$ undersampled signal with zero interpolation is composed of m copies of the DFT of the squeezed undersampled signal, $g[n]$. Furthermore, since $g[n]$ is real (optical intensity at the photodetector), the amplitude of DFT($g[n]$) has even symmetry, meaning that half of the amplitude of DFT($g[n]$) contains all the information to fully characterize the amplitude of DFT($g_0[n]$); this feature justifies the copy/flipping and concatenation operation shown in Figs. S1-S2.

We performed a quantitative comparison between the above detailed spectral squeezing method and the zero interpolation method on 2× and 3× undersampled spectral data. The image reconstruction results are summarized in Fig. S2. Through visual inspection, one can conclude that the undersampled, squeezed spectral data can be used to reconstruct the same high-quality OCT images that are achieved using the

undersampled images processed using zero interpolation. To further compare the above methods quantitatively, we calculated the average PSNR and SSIM values for 13,131 test image patches, which revealed very similar results using the two methods (see Table S1).

Although the undersampled squeezed OCT image contains all the needed information, directly using it as the network input (without the copy/flipping and concatenation operations) leads to spatial artifacts at the network output (see Fig. S3) since this represents a more complex and challenging learning and inference task for the U-net structured model that we have employed in our work.

In summary, we demonstrated the reconstruction of high-quality OCT images by using undersampled squeezed spectral data. With *m*-fold undersampled and squeezed spectral data, the computation time of an FFT operation can be decreased approximately by *m*-fold, and since the additional computational time needed for the flipping and concatenation operations is negligible compared to an FFT operation, the pre-processing method using squeezed, undersampled spectral data requires approximately (1/*m*)-*th* of the conventional OCT reconstruction time.

DL-OCT performance on different types of tissue

To further test the robustness of DL-OCT, we investigated its performance on different types of tissue imaged by an SS-OCT system, which had a central wavelength of ~1300 nm, a sweep range of ~100 nm, and an incident power of ~18 mW. The axial and transverse resolutions of the system have been characterized as ~12 µm and ~10 µm, respectively, in air. For this new dataset, a sample area of 5.5 mm × 5.5 mm × 7.27 mm (X, Y, Z) was imaged. Each raw A-scan consisted of 2560 spectral data points that were sampled linearly in the wavenumber domain by a k-clock on the SS-OCT system. Each B-scan consisted of 1000 A-scans, and each sample volume consisted of 1000 B-scans. After 2× undersampling, zero interpolation and OCT reconstruction, the resulting 2× undersampled images were partitioned into patches of 1280×320 pixels before being fed into the reconstruction neural network.

In our data acquisition, 7 human subjects and 4 mouse subjects were involved. For human subjects, 6 different types of samples, including finger, nail, palm, wrist, limbus of eye, and anterior chamber of eye, were imaged for each subject (a total of 42 human samples). For mouse subjects, the eye for each subject was imaged (resulting in a total of 4 mouse samples). In total, this dataset contains 7 different types of human/mouse tissue with 46 samples. To separate the network training and blind testing, one sample for each type of human/mouse tissue was reserved for blind testing, and the remaining 39 samples were mixed and used for training. Stated differently, for each human tissue type, one subject (and his/her sample) was left out for blind testing; similarly, one mouse subject was left out for blind testing.

The results of our blind testing for different types of tissue are summarized in Fig. S4. When visually inspected, one can see that DL-OCT successfully removed the spatial aliasing artifacts for all these different types of tissue. Quantitatively, DL-OCT achieved an average 28.7683 dB PSNR and 0.7239 SSIM on 7,000 full-frame test images.

Supplementary Table

Table. S1 Comparison of PSNR and SSIM values between the network output images and the corresponding ground truth SS-OCT images for different pre-processing methods.

Processing method	PSNR (dB)		SSIM	
	Average	Standard deviation	Average	Standard deviation
2× undersampled, squeezed	24.5937	1.4333	0.4449	0.0290
3× undersampled, squeezed	23.4407	1.3072	0.3145	0.0273
2× zero interpolated	24.6580	1.3225	0.4391	0.0320
3× zero interpolated	23.4012	1.2536	0.3080	0.0259

Supplementary Figures

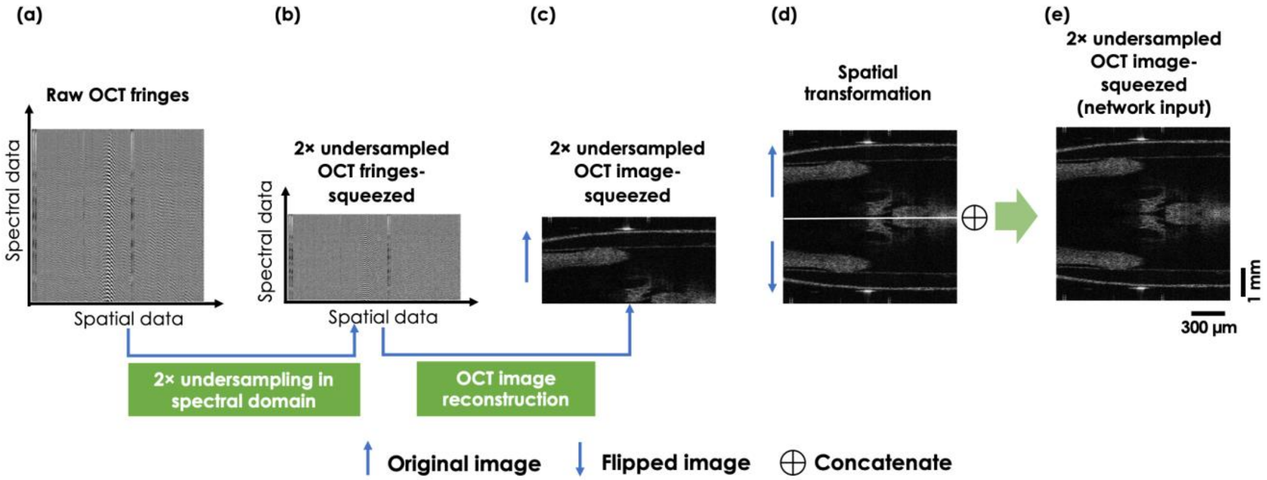

Figure S1. Schematic of the pre-processing method for DL-OCT using 2× undersampled, squeezed spectral data.

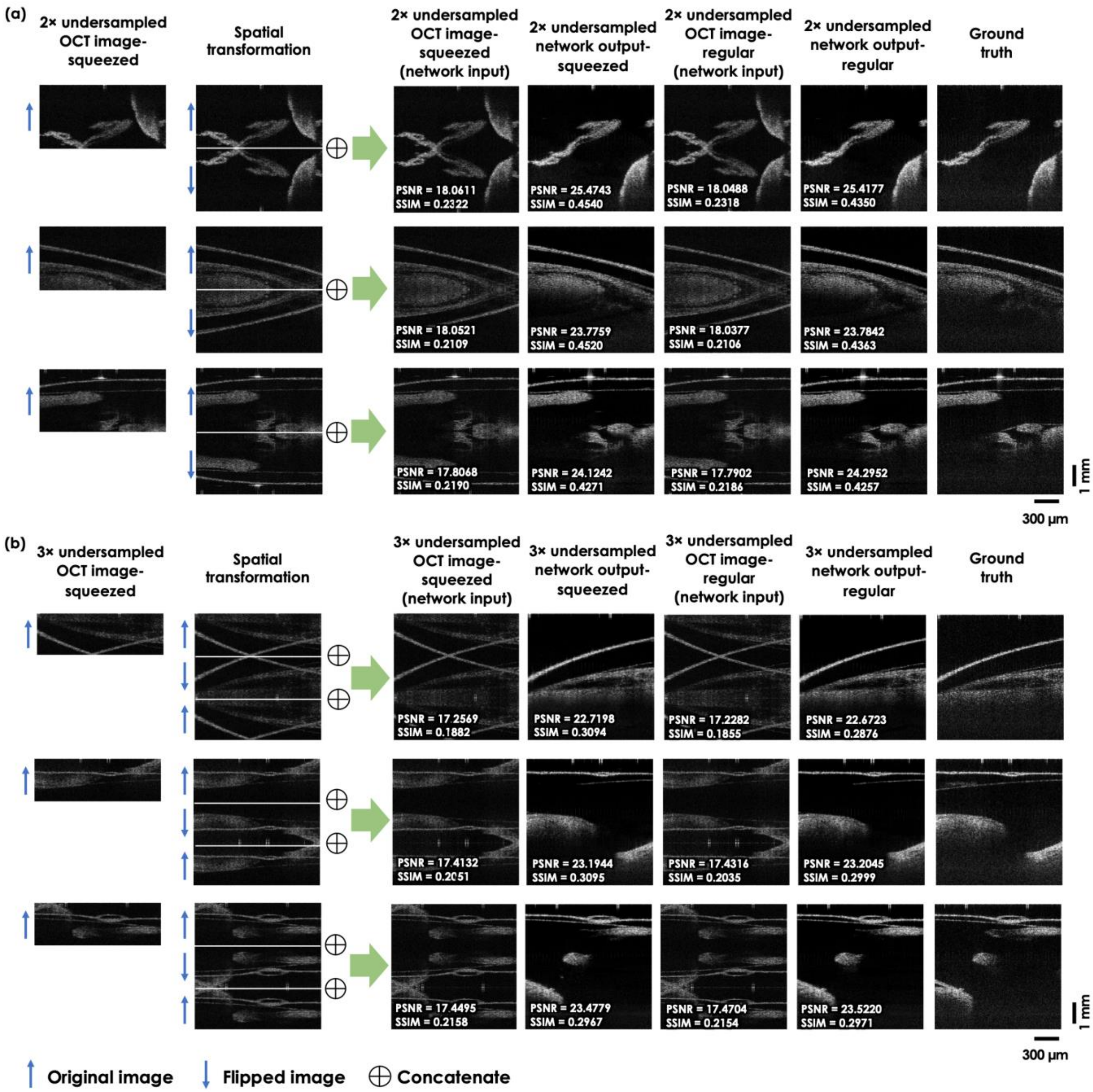

Figure S2. Comparison of blind testing results using undersampled, squeezed spectral data and undersampled spectral data with zero interpolation. (a) Comparison of 2× spectral undersampling results. (b) Comparison of 3× spectral undersampling results. PSNR and SSIM values are displayed for each one of these fields-of-view.

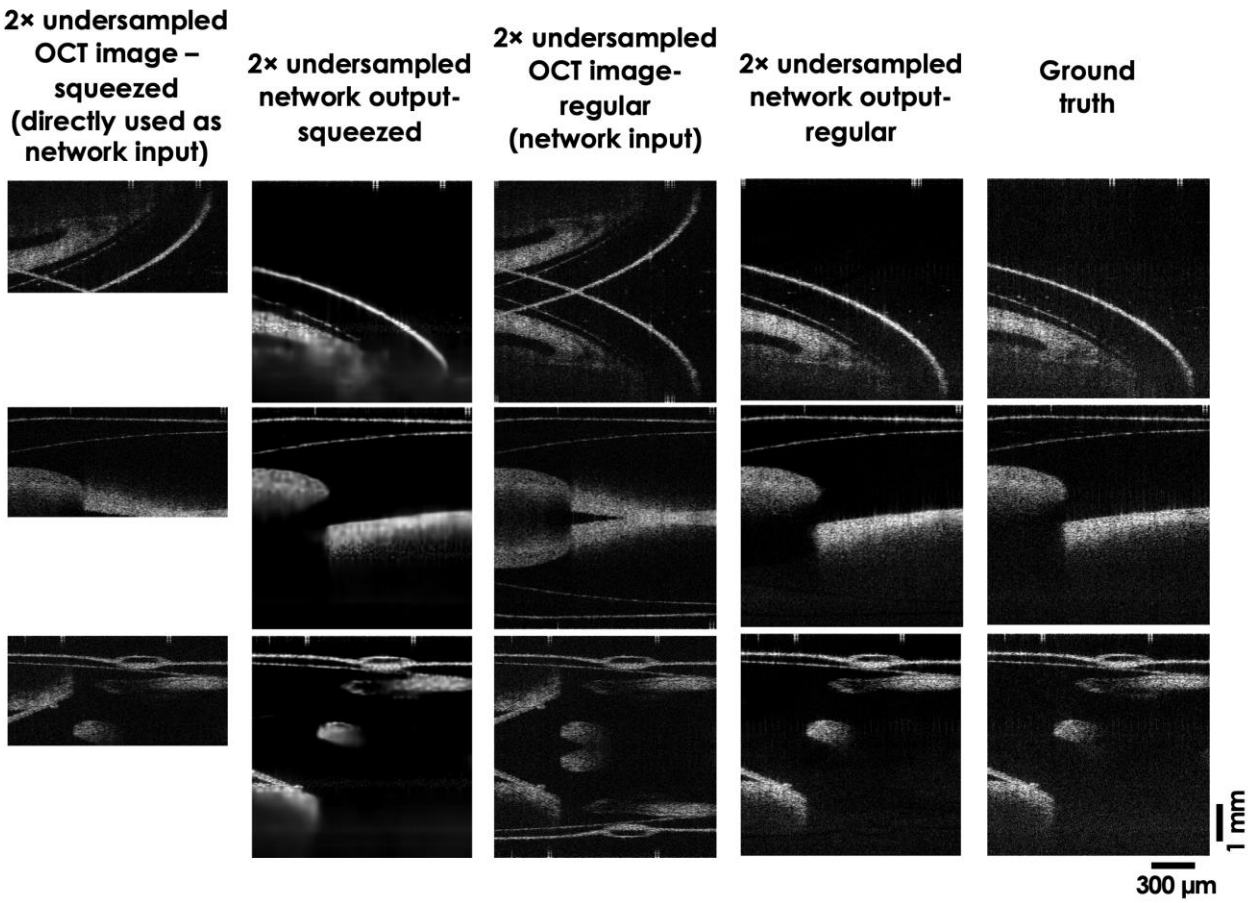

Figure S3. Comparison of blind testing results obtained using networks trained with 2× undersampled, squeezed spectral data *without* any spatial transformations vs. 2× undersampled spectral data with zero interpolation. These results confirm the significance of the spatial transformations reported in Fig. S2 in terms of the final image reconstruction quality. Although the undersampled, squeezed image contains all the needed information, directly using it as the network input (without the copy/flipping and concatenation operations reported in Fig. S2) leads to spatial artifacts at the network output (shown in the second column).

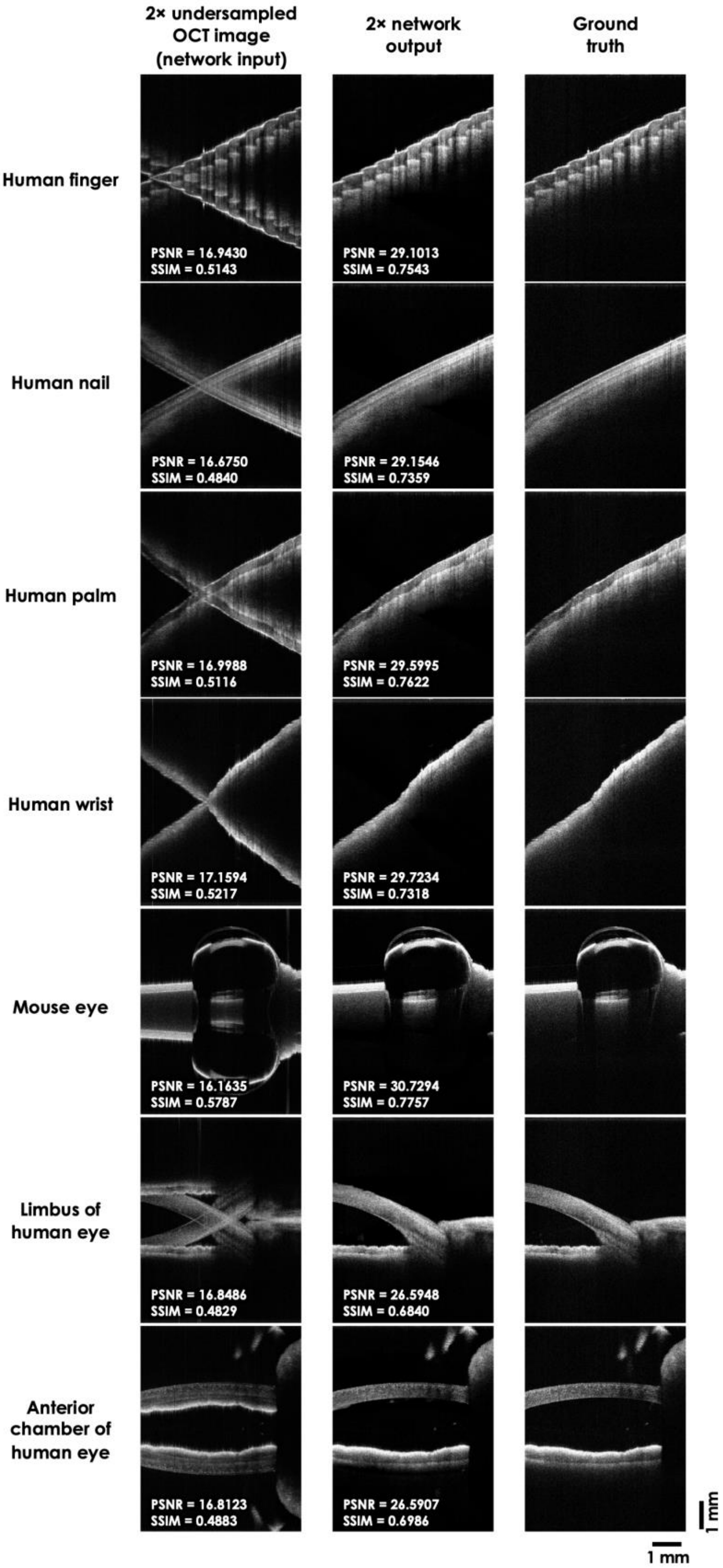

Figure S4. Blind testing performance of the DL-OCT on seven different types of tissue. PSNR and

SSIM values are also displayed for each one of these fields-of-view.

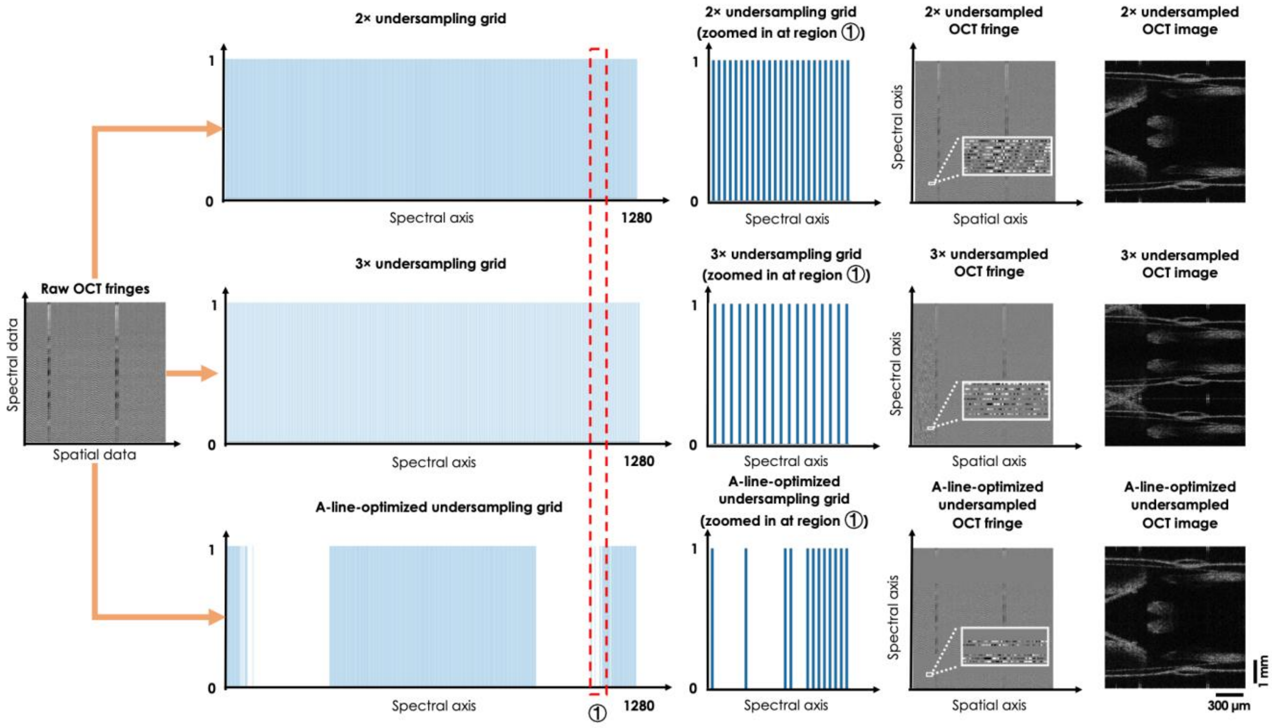

Figure S5. Comparison of DL-OCT network input workflow using 2× spectral undersampling, 3× spectral undersampling and A-line-optimized spectral undersampling methods. Raw OCT fringes were processed with 2× undersampling grid (N_{spec} = 640), 3× undersampling grid (N_{spec} = 427) and A-line-optimized undersampling grid (N_{spec} = 407), respectively. The resulting undersampled spectral fringes were then reconstructed to yield the corresponding undersampled OCT images, which served as the network input images (to be reconstructed). The A-line-optimized undersampling grid shown in the second column is a result of the A-line-optimized spectral undersampling method described in Fig. 8 of the main text.
